# Palmitoylation of *Plasmodium* alveolins promotes cytoskeletal function

**DOI:** 10.1016/j.molbiopara.2017.02.003

**Published:** 2017-04

**Authors:** Annie Z. Tremp, Fatimah S. Al-Khattaf, Johannes T. Dessens

**Affiliations:** Department of Pathogen Molecular Biology, Faculty of Infectious and Tropical Diseases, London School of Hygiene and Tropical Medicine, Keppel Street, London WC1E 7HT, UK

**Keywords:** Intermediate filament, *Plasmodium berghei*, Acyl-biotin exchange, Motility

## Abstract

S-palmitoylation is a post-translational lipid modification that is widespread among *Plasmodium* proteins and essential for parasite development. Little is known about the contribution of palmitoylation to the function of individual parasite molecules and structures. Alveolins are major components of the subpellicular network (SPN), a cortical cytoskeleton primarily involved in providing mechanical strength to the cell. We show here that the alveolin IMC1c is palmitoylated on a conserved cysteine motif, and that non-palmitoylated IMC1c displays normal expression, stability and trafficking. However, mutant parasites exhibit reduced osmotic stress resistance and tensile strength. These findings support the hypothesis that alveolin palmitoylation enhances cytoskeletal function by strengthening the connection between the SPN and the adjoining inner membrane complex via lipid anchoring.

## Highlights

• The alveolin IMC1c is palmitoylated on a conserved cysteine motif.
• Non-palmitoylated IMC1c exhibits normal stability and trafficking.
• Palmitoylation of *Plasmodium* alveolins enhances tensile strength.

## Graphical abstract

**Figure:**
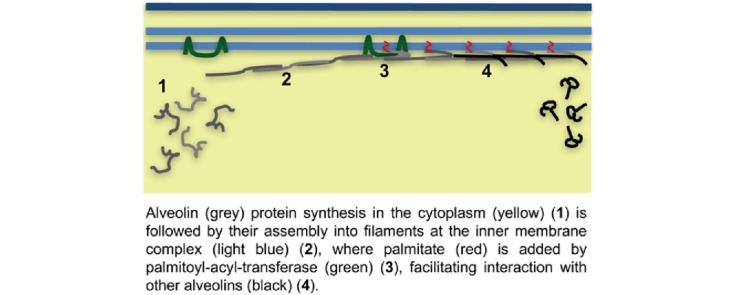

## 1 Introduction

Three invasive life stages: the ookinete, sporozoite and merozoite, feature among the many different developmental forms of the malaria parasite. These so-called ‘zoites’ are characterised by having specialised secretory organelles (e.g. rhoptries and micronemes) as well as a specialised cortical structure termed the pellicle, both of which are adaptations required for their motile and invasive properties. A double membrane structure termed the inner membrane complex (IMC) situated directly underneath the plasma membrane defines the pellicle [1], [2], [3]. In addition, a network of intermediate filaments termed the subpellicular network (SPN), which supports the pellicle membranes and provides mechanical strength to the cell [4], is located on the cytoplasmic face of the IMC. The main components of the SPN include a family of proteins termed alveolins [4], [5]. In the genus *Plasmodium*, 13 conserved and syntenic alveolin family members have been identified that are differentially expressed among the three zoites stages [6], [7]. It has been shown in the rodent malaria species *Plasmodium berghei* that disruption of alveolins is accompanied by reduced tensile strength of the zoite stages in which they are expressed [5], [7], [8], [9], [10]. *Plasmodium* alveolins have also been shown to have roles in parasite morphogenesis and gliding motility [5], [8], [9], [10]. Alveolins are characterised by the presence of one or more highly conserved domains composed of tandem repeat sequences [6], [11], [12]. In addition to these conserved ‘alveolin domains’, a subset of the alveolins also possess conserved cysteine motifs close to their amino- or carboxy-terminus, which are predicted to act as substrates for post-translational S-palmitoylation [13], [14].

S-palmitoylation is a post-translational thioester linkage of the 16-carbon fatty acid palmitate to cysteine residues [15], a reaction that is catalysed by palmitoyl-S-acyl-transferases (PATs). PATs are identifiable from having a Asp-His-His-Cys (DHHC) motif within a cysteine-rich domain, and at least 12 distinct putative PAT-encoding genes have been identified in *Plasmodium* spp. [16]. Localisation studies indicate that the subcellular distribution of many PATs is restricted to distinct compartments including endoplasmic reticulum, IMC and rhoptries [16]. S-palmitoylation in *Plasmodium* affects over 400 proteins and is essential for development of liver, blood and mosquito stages of the parasite [17], [18], [19], [20]. However, little is known about the specific contributions of this lipid modification to the function of individual parasite proteins. Furthermore, most palmitoylation sites are poorly predictable. This study was aimed at investigating the potential contribution of palmitoylation to the function of the alveolin IMC1c in *P. berghei* (*Pb*IMC1c; PBANKA_120200). *Pb*IMC1c and its *Plasmodium* orthologues have a single conserved cysteine motif at their carboxy-terminus that is predicted to act as a palmitoylation site [13]. In *Plasmodium falciparum*, the protein was detected in the palmitoylated proteome of asexual blood stages [20] indicating that it is indeed palmitoylated. *Pb*IMC1c is expressed in all three zoites where it displays a cortical localisation consistent with that of the SPN, and is essential for asexual blood stage parasite development [21].

## 2 Materials and methods

### 2.1 Animal use

All laboratory animal work was subject to ethical review by the London School of Hygiene and Tropical Medicine, and was approved by the United Kingdom Home Office. Animal experiments were carried out in accordance with the United Kingdom Animals (Scientific Procedures) Act 1986 implementing European Directive 2010/63 for the protection of animals used for experimental purposes. Experiments were conducted in 6–8 weeks old female CD1 mice, specific pathogen free and maintained in filter cages. Animal welfare was assessed daily and animals were humanely killed upon reaching experimental or humane endpoints. Mice were infected with parasites by intraperitoneal injection, or by infected mosquito bite on anaesthetised animals. Parasitemia was monitored regularly by collecting of a small drop of blood from a superficial tail vein. Drugs were administered by intraperitoneal injection or where possible were supplied in drinking water. Parasitised blood was harvested by cardiac bleed under general anaesthesia without recovery.

### 2.2 Generation of transgenic parasite lines

**Fig. 1:**
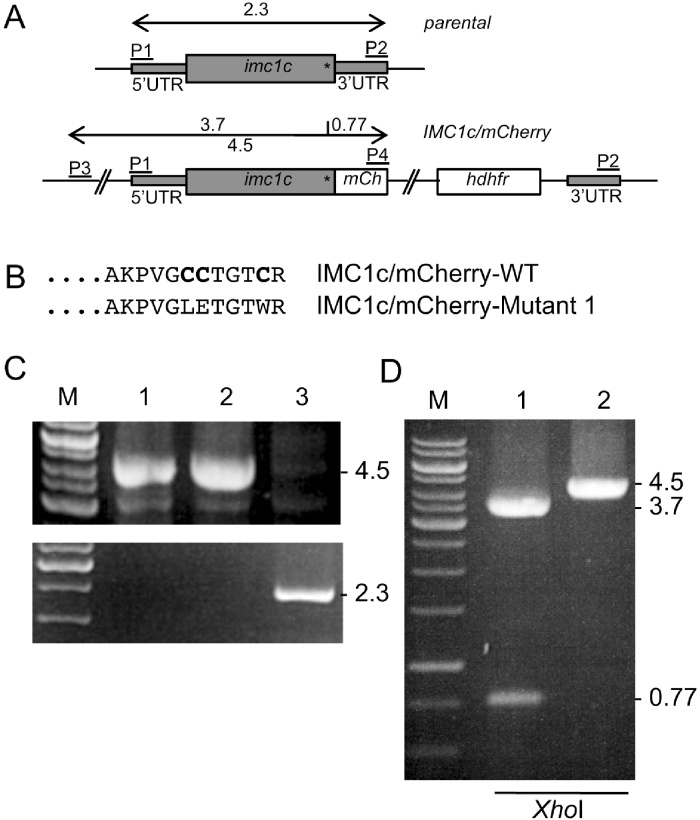
Generation and genetic analyses of mCherry-tagged *Pb*IMC1c parasite lines. A: Schematic diagram of the unmodified (*parental*) and modified (IMC1c/mCherry) *imc1c* alleles. The *imc1c* gene is indicated with coding sequence (wide grey bars) and 5′ and 3′ untranslated regions (UTRs, narrow grey bars). Also indicated is the relative positions of the terminal cysteine motif (asterisk), the mCherry module (*mCh*); the hDHFR selectable marker gene cassette (*hdhfr*); the *Xho*I site corresponding to the cysteine motif mutation in IMC1c/mCherry-Mutant 1; and primers used for diagnostic PCR amplification (P1-P4). B: Amino acid sequences of the carboxy-terminal sequences of *Pb*IMC1c in parasite lines IMC1c/mCherry-WT and IMC1c/mCherry-Mutant 1. The conserved cysteines are marked in bold. C: PCR with primers P3 and P4 across the 5′ integration site diagnostic for the presence of the mCherry-tagged *pbimc1c* alleles (top panel), and PCR with primers P1 and P2 diagnostic for the presence/absence of the unmodified *pbimc1c* allele (bottom panel), carried out on genomic DNA from clonal populations of parasite line IMC1c/mCherry-Mutant 1 (lane 1), IMC1c/mCherry-WT (lane 2) and parental parasites (lane 3). D: *Xho*I restriction enzyme digestion of the PCR amplicon from C, diagnostic for the presence/absence of the carboxy-terminal cysteine motif mutation.

Plasmid pLP-IMC1c/mCherry [21] was PCR-amplified with primers IMC1C-mut1-F (CTCGAGACAGGTACATGGAGAAGCTTAGGGGCCCTC) and IMC1C-Mut1-R (CCATGTACCTGTCTCGAGTCCAACTGGTTTAGCTTCTTCA) and circularised by in-fusion to give plasmid pLP-IMC1c/mCherry-Mutant 1. The site-directed mutagenesis was designed to substitute the three conserved cysteine residues at the carboxy terminus, and at the same time to introduce a diagnostic *Xho*I restriction site at the site of the mutation (Fig. 1AB). The resulting targeting vector was digested with *Kpn*I and *Sac*II to remove the plasmid backbone prior to transfection of purified *P. berghei* schizonts as described [21]. Pyrimethamine-resistant parasites were selected and dilution cloned to give parasite line IMC1c/mCherry-Mutant 1.

### 2.3 Palmitoylation assay

Infected blood was harvested and parasites purified by red blood cell lysis, followed by three washes in phosphate buffered saline (PBS, pH 7.4) to remove cellular debris. Parasite pellets were dissolved in lysis buffer (50 mM Tris–HCl pH 7.2, 1% SDS, 1× protease inhibitor cocktail (PI, Sigma), 5 mM EDTA, 1 mM TCEP) and then treated with 40 mM N-ethylmaleimide (NEM) overnight at 4 °C with nutation to block free cysteines. The following day SDS, TCEP and NEM were removed from the samples by buffer exchange on Zeba spin desalting columns (Thermo Scientific) equilibrated with hydroxylamine (HAM) buffer (1× PBS pH7.4, 1× PI, 5 mM EDTA). Each sample was divided into two equal parts and to each was added an equal volume of HAM buffer supplemented with 2 mM EZ-link HPDP-biotin (Thermo Fisher Scientific) and either 100 mM HAM (labelled +HAM) or no HAM (labelled −HAM). Samples were incubated 2 h at room temperature to allow palmitate-biotin exchange, after which HAM and unbound HPDP-biotin were removed by buffer exchange on Zeba spin desalting columns equilibrated with HAM buffer supplemented with 0.1% Triton X-100. Streptavidin-agarose suspension (Sigma) was added and the mixture incubated for 1 h at room temperature with nutation to allow binding to and subsequent pulldown of the biotinylated protein fraction. Agarose beads were collected by centrifugation, twice washed with HAM buffer plus 1% Triton X-100, and finally resuspended in an equal volume of 2x SDS polyacrylamide gel electrophoresis sample buffer supplemented with 1% β-mercaptoethanol to release biotinylated protein from the streptavidin-agarose beads. Samples were heated at 70 °C for 10 min prior to fractionation through NuPage 4–12% Bis-Tris precast gels (Invitrogen).

### 2.4 Ookinete size measurements (footprint method)

We recently developed the footprint method for sporozoites to enable a quantitative determination of sporozoite size independent of cell shape that is easy to standardise [13]. This method was adapted here for ookinetes. Briefly, thin films of purified, cultured ookinetes were made on glass microscope slides and air dried. After methanol fixation, Giemsa-stained images of cells were captured by microscopy on Zeiss LSM510 inverted laser scanning confocal microscope. Subsequently, using Zeiss LSM image browser software the circumference of individual ookinetes was measured, and the surface area occupied (i.e. the footprint) calculated. Statistical analysis was carried out using two-tailed *t*-test.

### 2.5 Hypo-osmotic shock and cell viability assay

Ookinetes in PBS were subjected to hypo-osmotic shock of 0.5 × PBS by adding an equal volume of water, or of 0.4 × PBS by adding 1.5 volumes of water. After 5 min, normal osmotic conditions were restored by adding an appropriate amount of concentrated PBS. Cell viability was scored by fluorescence microscopy in the presence of 0.5% propidium iodide and 1% Hoechst 33258. Ookinetes whose nucleus stained positive for both propidium iodide and Hoechst were scored as non-viable, whereas ookinetes whose nucleus only stained positive for Hoechst were scored as viable. At least one hundred ookinetes were scored per treatment, and all values were normalised for cell death before hypo-osmotic shock.

## 3 Results

### 3.1 The cysteine motif of IMC1c is dispensable for parasite development

Parasite line IMC1c/mCherry-WT, which expresses full-length IMC1c fused to a carboxy-terminal red fluorescent protein (mCherry variant), was generated by double crossover homologous recombination (Fig. 1A). To study the function of the carboxy-terminal cysteine motif of *Pb*IMC1c and its predicted role as a palmitoylation site, a specific mutation substituting the three cysteines was introduced by site-directed mutagenesis (Fig. 1B). The mutation introduced a diagnostic *Xho*I restriction site at the same time. Diagnostic PCR across the 5′-integration site with primers IMC1c-long5′UTR-F (P3) (GGCTCTCAAATTCTTGGAAG) and pDNR-mCherry-R (P4) (AACGGGATCTTCTAGTTACTTGTACAGCTCGTCCATGC) gave rise to a 4.5 kb product, as was the case for the corresponding IMC1c/mCherry-WT line, demonstrating correct integration of the modified allele into the *imc1c* locus (Fig. 1C, top panel). Moreover, the PCR product amplified from parasite line IMC1c/mCherry-Mutant 1 possessed the unique *Xho*I recognition sequence confirming that the cysteine motif mutation was indeed present (Fig. 1D). The absence of the wildtype *imc1c* allele in the transgenic parasite lines was confirmed by diagnostic PCR with primers pDNR-IMC1c-F (P1) (ACGAAGTTATCAGTCGACGGTACCAAGTGCATTTAGTATGTTGTGGC) and IMC1c-3′R (P2) (TTAGAGCCGATTTTATCTTGTTACAC), amplifying an expected 2.3 kb fragment from unmodified parental parasites, but not from the clonal transgenic IMC1c/mCherry lines (Fig. 1C, bottom panel).

**Fig. 2:**
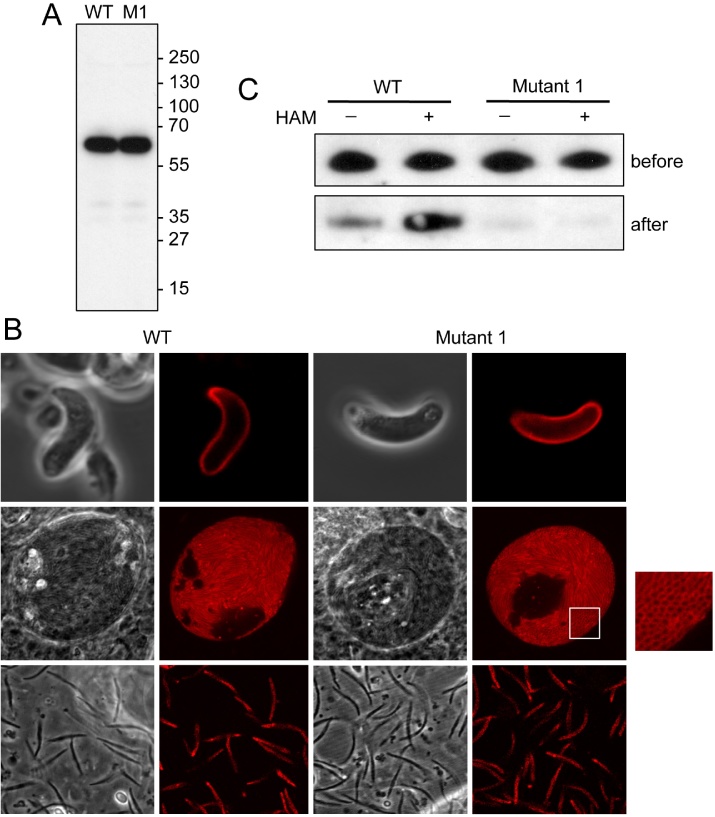
Phenotypic analyses of mCherry-tagged *PbI*MC1c parasite lines. A: Western blot using anti-mCherry antibodies of blood stage parasites from parasite line IMC1c/mCherry-WT (WT) and IMC1c/mCherry-Mutant 1 (M1). Positions of molecular weight markers (PageRuler™ Prestained Protein Ladder +) are shown on the right hand side. B: Confocal fluorescence and brightfield images of ookinetes (top), sporulating oocysts (middle) and sporozoites (bottom) of parasite lines IMC1a/mCherry-WT and Mutant 1. White square marks an area of the oocyst showing transversally cross-sectioned sporozoites (inset), clearly showing the cortical fluorescence. C: Western blot of blood stage parasite lysates of parasite lines IMC1c/mCherry-WT and Mutant 1 after acyl-biotin exchange in the absence (−HAM) or presence (+HAM) of hydroxylamine, *before* and *after* pull-down with streptavidin-agarose beads.

Parasite lines IMC1c/mCherry-WT and Mutant 1 developed normally in the mouse and when examined by UV microscopy displayed red fluorescence in the asexual blood stages and macrogametocytes similar to that reported for parasite line IMC1c/GFP that expresses GFP-tagged IMC1c [21] (data not shown). Consistent with this observation, western blot analysis of blood stage parasites of both parasite lines using anti-mCherry antibodies revealed a strong band of approximately 60 kDa, corresponding to the IMC1c::mCherry fusion proteins (Fig. 2A). In the mosquito, parasite line IMC1c/mCherry-Mutant 1 developed normally and based on visual assessment was indistinguishable from its wildtype counterpart in terms of ookinete and sporozoite shape, IMC1c expression level and distribution (Fig. 2B). Fluorescence was concentrated at the cortex of the zoites consistent with a localisation in the SPN (Fig. 2B). Both parasite lines formed comparable numbers of oocysts (mean ± sem 30 ± 13 for IMC1c/Mcherry-WT; 31 ± 9 for Mutant 1; *P* = 0.97; *n* = 20) and were readily transmitted to naive mice by sporozoite-infected mosquito bite. These results demonstrate that the carboxy-terminal cysteine motif of *Pb*IMC1c is dispensable for parasite development in the mouse and mosquito.

### 3.2 IMC1c is palmitoylated on its carboxy-terminal cysteine motif

Palmitoylation of IMC1c was assessed biochemically using a modification of the acyl-biotin exchange method [22]. Western analysis using anti-mCherry antibodies detected IMC1c::mCherry signal in the HAM-treated IMC1c/mCherry-WT sample that was markedly stronger than in the HAM-negative control relative to pre-pulldown signals (Fig. 2C). In the absence of HAM, palmitate-biotin exchange does not occur, and thus the increased signal in the HAM-positive sample indicates that *Pb*IMC1c is palmitoylated (the signal in the HAM-negative sample is likely the result of some incomplete blocking of free cysteine residues by the earlier NEM treatment). In the IMC1c/mCherry-Mutant 1 samples, IMC1c::mCherry signals were barely detectable in either −HAM and +HAM treatments (Fig. 2C). Collectively, these results provide biochemical evidence that *Pb*IMC1c is palmitoylated on its carboxy-terminal cysteine motif.

### 3.3 Palmitoylation of IMC1c enhances cytoskeletal function

Although parasite development of Mutant 1 in host and vector was indistinguishable from that of its wildtype counterpart, the demonstrated involvement of alveolins in motility and tensile strength provision prompted us to test whether a phenotype might be associated with any of these properties. This was carried out with cultured ookinetes, which are easily accessible and for which reliable motility and tensile strength assays exist [8], [9], [23]. No detectable differences in parasite locomotion were observed: ookinete motility through Matrigel was comparable between the two parasite lines (mean ± sem 31.7 ± 3.3 μm per 10 min for WT; 33.8 ± 1.6 μm for Mutant 1; *n* = 20; *t*-test *P* = 0.68). Moreover, ookinetes from both lines displayed a meandering movement typical of helical gliding as described [10].

**Fig. 3:**
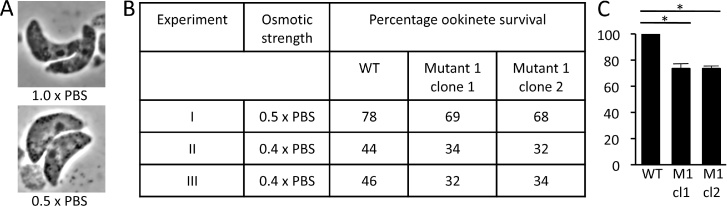
Sensitivity to hypo-osmotic shock of parasite lines IMC1c/mCherry-WT and IMC1c/mCherry-Mutant 1. A: Images of Giemsa-stained ookinetes in normal (1.0 × PBS) and hypo-osmotic (0.5 × PBS) conditions, showing swelling of the cells due to water uptake. B: Percentage ookinete survival after 5 min exposure to hypo-osmotic shock. At least one hundred ookinetes were scored per treatment, and all values were normalised for cell death before hypo-osmotic shock. C: Bar chart of ookinete viability after hypo-osmotic shock (0.4 × PBS) expressed as a percentage of the control parasite (IMC1c/mCherry-WT) value. Error bars represent standard error of the mean (*n* = 2). Statistically significant differences (*P* < 0.05, *t*-test) are indicated (*).

To assess ookinete tensile strength, cells were subjected to hypo-osmotic conditions, which forces the cells to draw in water and swell. The ability to withstand hypo-osmotic shock is routinely used as a relative measure of tensile strength [24], based on the fact that cells with reduced tensile strength swell more leading to increased cell death. Under normal osmotic conditions (1.0 × PBS), the mean size of ookinetes as determined by our footprint assay was indistinguishable between IMC1c/mCherry-WT and Mutant 1 parasites (mean ± sem 28.7 ± 0.63 μm^2^ for IMC1c/Mcherry-WT; 29.1 ± 0.55 μm^2^ for Mutant 1; *n* = 30; *P* = 0.67), confirming our observations of live cells (Fig. 2B). Ookinete size increased significantly (*P* < 0.0001) upon hypo-osmotic exposure (0.5 × PBS) in both parasite lines (mean ± sem 34.6 ± 0.56 μm^2^ for IMC1c/Mcherry-WT; 36.3 ± 0.61 μm^2^ for Mutant 1; *n* = 30), reflecting the uptake of water by the cells (Fig. 3A). Compared to the control parasite, Mutant 1 ookinetes displayed a greater and statistically significant size increase (*P* < 0.05) under these hypo-osmotic conditions, pointing to a reduction in their tensile strength. Indeed, this notion was supported by measurements of ookinete viability after hypo-osmotic shock: Exposure of cultured ookinetes for 5 min to 0.5 × PBS caused 22% cell death in the control parasite line IMC1c/mCherry-WT, which increased to approximately 31% in parasite line IMC1c/mCherry-Mutant 1 (Fig. 3B). More stringent hypo-osmotic shock (5 min exposure to 0.4 × PBS) resulted in higher, approximately 55% cell death in control parasites that increased to approximately 67% in Mutant 1 parasites (Fig. 3B). When expressed as a percentage of the control parasite values, Mutant 1 displayed on average 27% higher sensitivity in response to hypo-osmotic shock (Fig. 3C). These statistically significant differences in sensitivity were consistent across clones and biological replicates, indicating that Mutant 1 ookinetes possess reduced tensile strength as a consequence of lacking IMC1c palmitoylation.

## 4 Discussion

Cells possess broadly three types of cytoskeletal filaments: (i) microfilaments, composed predominantly of actin; (ii) microtubules, composed mainly of tubulin, and (iii) intermediate filaments (IFs). While metazoan proteins that form IFs (e.g. lamin, keratin, desmin) are structurally diverse, they share certain architectural features, the most important among these being a helical rod domain that is able to supercoil with the same domain in another IF molecule to form a coiled-coil structure. Formation of the coiled-coil dimer is fundamental to the filamentous features of these molecules. The coiled-coiling properties of the helical domain are conferred at the primary structure level by heptad repeats: tandem repeats of seven amino acids in a specific arrangement, although variations on this theme are common. While cytoskeletal IFs in metazoans, particularly in vertebrates, have long been studied, their existence in protists is not widely recognised. It is becoming increasingly evident, however, that protists may possess novel types of IF proteins [25], [26], with the caveat that direct experimental evidence for their IF-like properties is not currently available. Alveolins are an example of such novel putative IF proteins, and this study sheds further light on their function, in particular with regards to the contribution of post-translational palmitoylation.

The results of this study confirm that the carboxy-terminal cysteine motif of the alveolin IMC1c acts as a palmitoylation site. They also highlight the fact that palmitoylation of IMC1c is not required for protein stability, or for recruitment to the SPN and IMC targeting (Fig. 2B). *Pb*IMC1c contains no other predicted sites for post-translational lipidation, indicating that its core alveolin domain is solely sufficient for IMC targeting. This contrasts with palmitoylation of the IMC sub-compartment proteins (ISPs), which was shown to be necessary for correct IMC targeting of ISPs in both *Toxoplasma gondii* and *P. falciparum* [27], [28]. In sharp contrast to IMC1c, mutation of equivalent cysteine motifs in the sporozoite-expressed alveolin IMC1a in *P. berghei* were recently shown to have a marked adverse effect on the protein's stability and, consequently, on parasite fitness [13]. What might explain these differences? Using quantitative mass spectrometry, Jones and colleagues showed that palmitoylation of IMC1c in *P. falciparum* blood stage parasites is highly sensitive to the presence of the palmitoylation inhibitor 2-bromopalmitate (2-BMP) [20]. Given that absence of palmitoylation does not result in degradation of IMC1c, as shown in this study, its sensitivity to 2-BMP could point to a dynamic palmitoylation status involving cycles of palmitoylation and depalmitoylation. Another explanation is that the inhibitor was present before IMC1c was palmitoylated. Given that PATs are membrane-spanning enzymes and alveolins reside in the SPN, it is likely that alveolins are palmitoylated by IMC-resident PATs [19], [28]. Accordingly, palmitoylation would not occur until after SPN recruitment/IMC targeting of the alveolin. Contrary to IMC1a that is recruited to the SPN concomitant with pellicle formation [5], IMC1c is recruited late to the SPN after pellicle formation and zoite morphogenesis has happened [21]. By implication, palmitoylation of IMC1c in the blood stages would not take place until completion of cytokinesis in the schizonts, which could explain why 2-BMP is so effective at blocking this step. In either scenario (extensive palmitoyl cycling or delayed palmitoylation), IMC1c would spend a considerable part of its lifetime in a non-palmitoylated state and it makes sense therefore that it should be stable in the absence of this lipid modification. Conversely, the markedly reduced stability of IMC1a in response to losing cysteine motifs/palmitoylation sites [13] could be interpreted as the alveolin requiring a stable palmitoylation status and being palmitoylated soon after its synthesis. The presence and importance of palmitoyl cycling in *Plasmodium* remains to be determined.

We did not detect a phenotype of Mutant 1 parasites associated with motility. The SPN supports gliding motility most likely by tethering molecular motor components situated on the other side of the IMC via the GAPM proteins, a family of multi-pass membrane proteins that reside in the IMC and that interact with both alveolins and molecular motor proteins [29]. The lack of a motility phenotype combined with the apparent normal localisation of IMC1c in Mutant 1 (Fig. 2B) suggests that connecting the glideosome with the cytoskeleton can still take place efficiently in the absence of IMC1c palmitoylation, although it cannot be ruled out that a reduction in motility may have been too small to detect using our assay. The absence of IMC1c palmitoylation in Mutant 1 also did not discernibly affect zoite shape. There are two likely reasons for this: First, zoite morphogenesis is concurrent with pellicle formation, and because IMC1c is recruited to the SPN after pellicle formation [21], IMC1c is unlikely to influence this process. Second, tensile strength reduction does not automatically affect zoite morphogenesis, because these two processes are uncoupled [23].

**Fig. 4:**
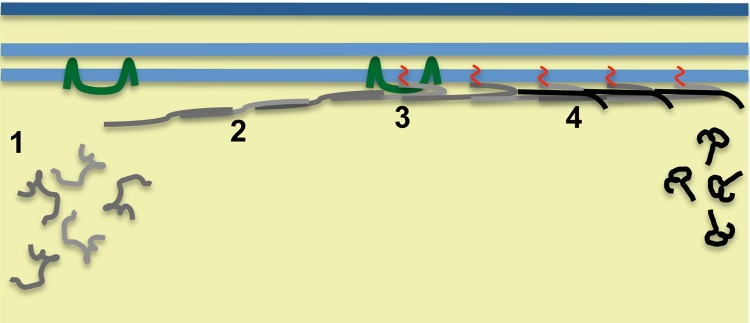
Model of alveolin synthesis, recruitment and assembly into the subpellicular network of a *Plasmodium* zoite to form a functional cytoskeleton. Depicted is a section of a cell showing plasma membrane (dark blue) and cytoplasm (yellow). The key steps indicated are: (1) Synthesis of alveolin proteins (grey) in the cytoplasm. (2) Targeting of the alveolins to the inner membrane complex (light blue) via the alveolin domain, and assembly into intermediate filaments. (3) Addition of palmitoyl lipid (red) to the alveolin by IMC-resident palmitoyl-acyl-transferase (green) and membrane anchoring. (4) Interaction with other alveolins (black) in response to palmitoylation. (For interpretation of the references to colour in this figure legend, the reader is referred to the web version of the article.)

Our findings did, however, reveal a role of IMC1c palmitoylation in mechanical strength (Fig. 3). Our data support the model that the SPN acts as an internal cytoskeletal basket that provides tensile strength to the cell, and indicate that this function is enhanced by lipid anchoring the SPN into the IMC through alveolin palmitoylation (Fig. 4). A tight association of the SPN with the IMC could directly enhance the tensile strength of the SPN, or alternatively alveolin palmitoylation and membrane association could promote interactions with other alveolins and SPN components. This, in turn, could lead to increased SPN rigidity. In this context it is important to note that palmitoylation is known to enhance protein interactions [15]. Cell rigidity of the ookinete is likely to be important when it escapes the blood meal in the midgut lumen and crosses the peritrophic matrix and midgut epithelium, a process that has been described to cause major cell constrictions [30], [31]. Accordingly, weakened ookinetes with reduced tensile strength could be less effective at these invasive processes, or more prone to damage during it. The same could apply to sporozoites when they invade mosquito or human tissues.

Repeated failure to disrupt *pbimc1c* indicates that this alveolin is essential for blood stage parasite development [21]. The lack of a strong phenotype for its cysteine mutant reported here therefore implies that palmitoylation of this alveolin only marginally enhances protein function and contributes only a small fitness gain to the parasite. This suggests that the essential nature of palmitoylation at a cellular level could be the result of accumulative small fitness gains at a molecular level, which are relatively minor when considered individually. With regards to tensile strength provision, the SPN contains multiple alveolins besides IMC1c, some of which may equally be palmitoylated [6], [13]. While the removal of palmitoylation from one alveolin may have only a modest impact on SPN function, when palmitoylation of multiple alveolins were blocked the effect is likely to be more profound. This notion is consistent with observations that parasite lines in which specific PATs have been disrupted, thus affecting palmitoylation of many substrate proteins at the same time, in many cases result in severe phenotypes [17], [18], [19].

## References

[1] Bannister L.H., Hopkins J.M., Fowler R.E., Krishna S., Mitchell G.H. (2000). A brief illustrated guide to the ultrastructure of *Plasmodium falciparum* asexual blood stages. Parasitol. Today.

[2] Morrissette N.S., Sibley L.D. (2002). Cytoskeleton of apicomplexan parasites. Microbiol. Mol. Biol. Rev..

[3] Santos J.M., Lebrun M., Daher W., Soldati D., Dubremetz J.F. (2009). Apicomplexan cytoskeleton and motors: key regulators in morphogenesis, cell division, transport and motility. Int. J. Parasitol..

[4] Mann T., Beckers C. (2001). Characterization of the subpellicular network, a filamentous membrane skeletal component in the parasite *Toxoplasma gondii*. Mol. Biochem. Parasitol..

[5] Khater E.I., Sinden R.E., Dessens J.T. (2004). A malaria membrane skeletal protein is essential for normal morphogenesis, motility, and infectivity of sporozoites. J. Cell Biol..

[6] Al-Khattaf F.S., Tremp A.Z., Dessens J.T. (2015). *Plasmodium* alveolins possess distinct but structurally and functionally related multi-repeat domains. Parasitol. Res..

[7] Kaneko I., Iwanaga S., Kato T., Kobayashi I., Yuda M. (2015). Genome-wide identification of the target genes of AP2-O, a *Plasmodium* AP2-family transcription factor. PLoS Pathog..

[8] Tremp A.Z., Khater E.I., Dessens J.T. (2008). IMC1b is a putative membrane skeleton protein involved in cell shape, mechanical strength, motility, and infectivity of malaria ookinetes. J. Biol. Chem..

[9] Tremp A.Z., Dessens J.T. (2011). Malaria IMC1 membrane skeleton proteins operate autonomously and participate in motility independently of cell shape. J. Biol. Chem..

[10] Volkmann K., Pfander C., Burstroem C., Ahras M., Goulding D., Rayner J.C. (2012). The alveolin IMC1h is required for normal ookinete and sporozoite motility behaviour and host colonisation in *Plasmodium berghei*. PLoS ONE.

[11] Gould S.B., Tham W.H., Cowman A.F., McFadden G.I., Waller R.F. (2008). Alveolins, a new family of cortical proteins that define the protist infrakingdom Alveolata. Mol. Biol. Evol..

[12] El-Haddad H., Przyborski J.M., Kraft L.G., McFadden G.I., Waller R.F., Gould S.B. (2013). Characterization of TtALV2, an essential charged repeat motif protein of the *Tetrahymena thermophila* membrane skeleton. Eukaryot. Cell.

[13] Al-Khattaf F.S., Tremp A.Z., El-Houderi A., Dessens J.T. (2017). The *Plasmodium* alveolin IMC1a is stabilised by its terminal cysteine motifs and facilitates sporozoite morphogenesis and infectivity in a dose-dependent manner. Mol. Biochem. Parasitol..

[14] Anderson-White B.R., Ivey F.D., Cheng K., Szatanek T., Lorestani A., Beckers C.J. (2011). A family of intermediate filament-like proteins is sequentially assembled into the cytoskeleton of *Toxoplasma gondii*. Cell. Microbiol..

[15] Linder M.E., Deschenes R.J. (2007). Palmitoylation: policing protein stability and traffic. Nat. Rev. Mol. Cell Biol..

[16] Frenal K., Tay C.L., Mueller C., Bushell E.S., Jia Y., Graindorge A. (2013). Global analysis of apicomplexan protein S-acyl transferases reveals an enzyme essential for invasion. Traffic.

[17] Santos J.M., Kehrer J., Franke-Fayard B., Frischknecht F., Janse C.J., Mair G.R. (2015). The *Plasmodium* palmitoyl-S-acyl-transferase DHHC2 is essential for ookinete morphogenesis and malaria transmission. Sci. Rep..

[18] Santos J.M., Duarte N., Kehrer J., Ramesar J., Avramut M.C., Koster A.J. (2016). Maternally supplied S-acyl-transferase is required for crystalloid organelle formation and transmission of the malaria parasite. Proc. Natl. Acad. Sci. U.S.A..

[19] Hopp C.S., Balaban A.E., Bushell E., Billker O., Rayner J.C., Sinnis P. (2016). Palmitoyl transferases have critical roles in the development of mosquito and liver stages of *Plasmodium*. Cell. Microbiol..

[20] Jones M.L., Collins M.O., Goulding D., Choudhary J.S., Rayner J.C. (2012). Analysis of protein palmitoylation reveals a pervasive role in *Plasmodium* development and pathogenesis. Cell Host Microbe.

[21] Tremp A.Z., Al-Khattaf F.S., Dessens J.T. (2014). Distinct temporal recruitment of *Plasmodium* alveolins to the subpellicular network. Parasitol. Res..

[22] Wan J., Roth A.F., Bailey A.O., Davis N.G. (2007). Palmitoylated proteins: purification and identification. Nat. Protoc..

[23] Tremp A.Z., Carter V., Saeed S., Dessens J.T. (2013). Morphogenesis of *Plasmodium* zoites is uncoupled from tensile strength. Mol. Microbiol..

[24] Menke A., Jockusch H. (1991). Decreased osmotic stability of dystrophin-less muscle cells from the mdx mouse. Nature.

[25] Koreny L., Field M.C. (2016). Ancient eukaryotic origin and evolutionary plasticity of nuclear lamina. Genome Biol. Evol..

[26] Preisner H., Karin E.L., Poschmann G., Stuhler K., Pupko T., Gould S.B. (2016). The cytoskeleton of parabasalian parasites comprises proteins that share properties common to intermediate filament proteins. Protist.

[27] Beck J.R., Rodriguez-Fernandez I.A., Cruz de Leon J., Huynh M.H., Carruthers V.B., Morrissette N.S. (2010). A novel family of Toxoplasma IMC proteins displays a hierarchical organization and functions in coordinating parasite division. PLoS Pathog..

[28] Wetzel J., Herrmann S., Swapna L.S., Prusty D., John Peter A.T., Kono M. (2015). The role of palmitoylation for protein recruitment to the inner membrane complex of the malaria parasite. J. Biol. Chem..

[29] Bullen H.E., Tonkin C.J., O’Donnell R.A., Tham W.H., Papenfuss A.T., Gould S. (2009). A novel family of Apicomplexan glideosome-associated proteins with an inner membrane-anchoring role. J. Biol. Chem..

[30] Vernick K.D., Fujioka H., Aikawa M. (1999). *Plasmodium gallinaceum*: a novel morphology of malaria ookinetes in the midgut of the mosquito vector. Exp. Parasitol..

[31] Han Y.S., Thompson J., Kafatos F.C., Barillas-Mury C. (2000). Molecular interactions between *Anopheles stephensi* midgut cells and *Plasmodium berghei*: the time bomb theory of ookinete invasion of mosquitoes. EMBO J..

